# Supplementary material for: The interactome of intact mitochondria by cross-linking mass spectrometry provides evidence for coexisting respiratory supercomplexes
Source: Mol Cell Proteomics. 2017 Dec 8;17(2):216–32. doi: 10.1074/mcp.RA117.000470 (PMC5795388; doi:10.1074/mcp.RA117.000470)
Supplement: Supplemental Data [file supp_RA117.000470_133922_0_supp_23978_fzffwf.zip › spectra_annotation/mito_DR_spectra_annotation/25-1-4-1-7-1.pdf]

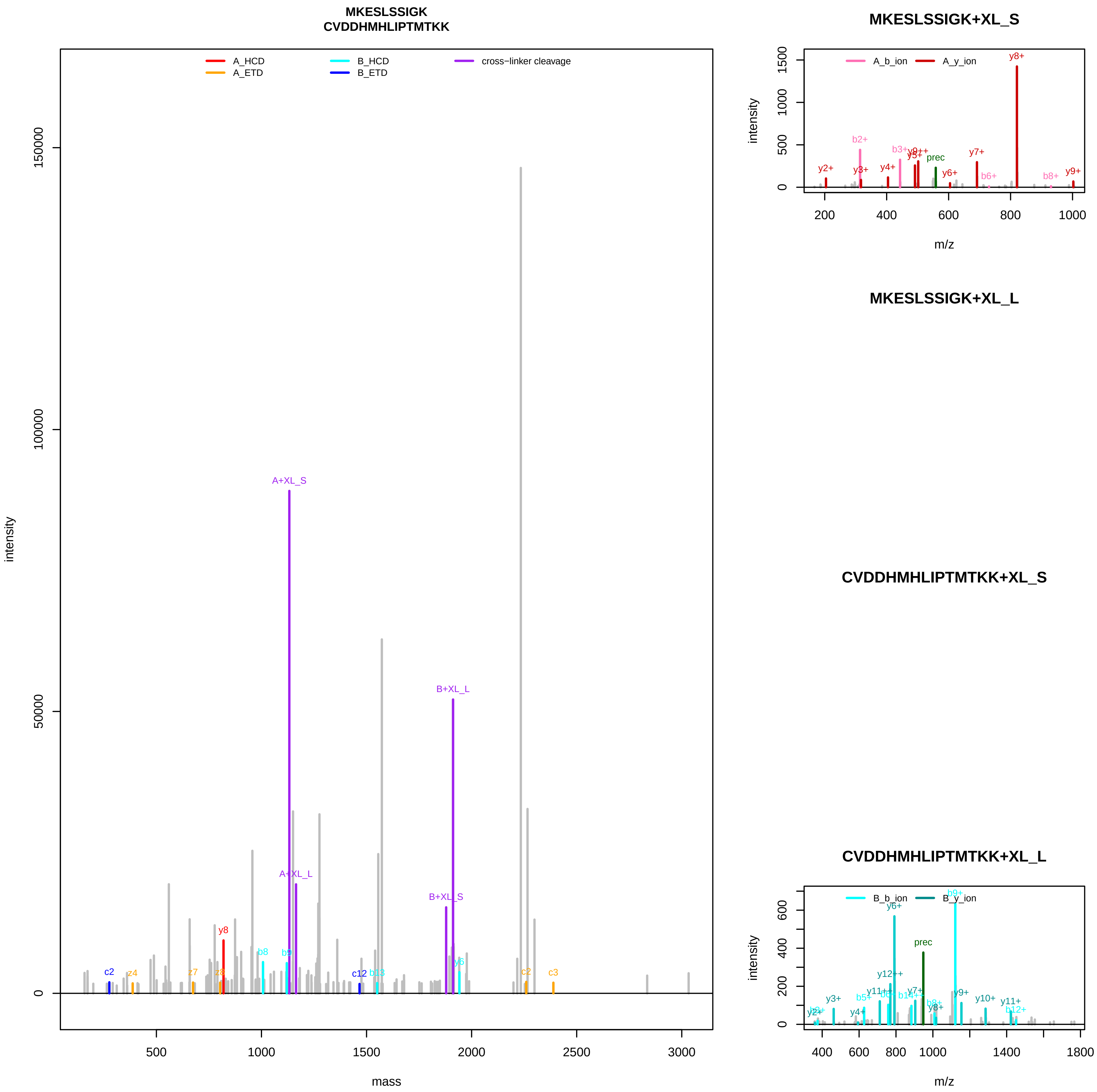
MKESLSSIGK
CVDDHMHLIPTMTKK
A_HCD
A_ETD
B_HCD
B_ETD
cross−linker cleavage
A+XL_S
A+XL_L
B+XL_L
B+XL_S
y8
b8
b5
c2
z4
z7
z8
c12
b13
y6
c2
c3
intensity
mass
0
50000
100000
150000
500
1000
1500
2000
2500
3000
MKESLSSIGK+XL_S
A_b_ion
A_y_ion
y8+
b2+
b3+
y9++
y5+
prec
y7+
y2+
y3+
y4+
y6+
b6+
b8+
y9+
intensity
m/z
0
500
1000
1500
200
400
600
800
1000
MKESLSSIGK+XL_L
CVDDHMHLIPTMTKK+XL_S
CVDDHMHLIPTMTKK+XL_L
B_b_ion
B_y_ion
b9+
y6+
prec
y12++
y11++
y7+
b14++
b5+
b6+
y3+
y4+
y2+
b3+
b8+
y8+
y9+
y10+
y11+
b12+
intensity
m/z
0
200
400
600
400
600
800
1000
1400
1800
